# DNA Barcoding: Promise and Pitfalls

**DOI:** 10.1371/journal.pbio.0020354

**Published:** 2004-09-28

**Authors:** Craig Moritz, Carla Cicero

In this issue of *PLoS Biology,* Hebert et al. (2004) have set out to test the resolution and performance of “DNA barcoding,” using a single mtDNA gene, cytochrome *c* oxidase I (COI), for a sample of North American birds. Before turning to details of this study, it is useful as context to consider the following questions: What is DNA barcoding, and what does it promise? What is new about it? Why is it controversial? What are the potential pitfalls?

Put simply, the intent of DNA barcoding is to use large-scale screening of one or a few reference genes in order to (i) assign unknown individuals to species, and (ii) enhance discovery of new species (Hebert et al. 2003; Stoeckle 2003). Proponents envisage development of a comprehensive database of sequences, preferably associated with voucher specimens representing described species, against which sequences from sampled individuals can be compared. Given the long history of use of molecular markers (e.g., allozymes, rDNA, and mtDNA) for these purposes (Avise 2004), there is nothing fundamentally new in the DNA barcoding concept, except increased scale and proposed standardization. The former is inevitable. Standardization, i.e., the selection of one or more reference genes, is of proven value in the microbial community and in stimulating large-scale phylogenetic analyses, but whether “one gene fits all” is open to debate.

Why, then, all the fuss? Initial reactions to the DNA barcoding concept have ranged from unbridled enthusiasm, especially from ecologists (Janzen 2004), to outright condemnation, largely from taxonomists (e.g., see the February 2003 issue of *Trends in Ecology and Evolution*). The former view reflects a real need to connect different life history stages and to increase the precision and efficiency of field studies involving diverse and difficult-to-identify taxa. The criticisms are mainly in response to the view that single-gene sequences should be the primary identifier for species (“DNA taxonomy”; Tautz et al. 2002; see also Blaxter 2004). At least for the macrobiota, the DNA barcoding community has moved away from this to emphasize the importance of embedding any large-scale sequence database within the existing framework and practice of systematics, including the importance of voucher specimens and of integrating molecular with morphological characters. Another point of contention—that DNA barcodes have limited phylogenetic resolution—arises from confusion about the scope of inference. At best, single-gene assays can hope to identify an individual to species or reveal inconsistencies between molecular variation and current perceptions of species boundaries. DNA barcoding should not be confused with efforts to resolve the “tree of life.” It should connect with and benefit from such projects, but resolving phylogeny at scales from species to major eukaryotic clades requires a very different strategy for selecting genes. Indeed, the very characteristic that makes the COI gene a candidate for high-throughput DNA barcoding—highly constrained amino acid sequence and thus broad applicability of primers (Hebert et al. 2003)—also limits its information content at deeper phylogenetic levels (e.g., Russo et al. 1996; Zardoya and Meyer 1996; Naylor and Brown 1997). Finally, while superficially appealing, the very term DNA barcoding is unfortunate, as it implies that each species has a fixed and invariant characteristic—like a barcode on a supermarket product. As evolutionary biologists, we should question this analogy.

In evaluating the promise and pitfalls of DNA barcoding, we need to separate the two areas of application: molecular diagnostics of individuals relative to described taxa, and DNA-led discovery of new species. Both are inherently phylogenetic and rely on a solid taxonomic foundation, including adequate sampling of variation within species and inclusion of all previously described extant species within a given genus. Accurate diagnosis depends on low intraspecific variation compared with that between species, such that a short DNA sequence will allow precise allocation of an individual to a described taxon. The extensive literature on mtDNA phylogeography (Avise 2000) indicates that this condition often holds, although there are exceptions. Furthermore, within many species there is sufficient structure that it will be possible to allocate an individual to a particular geographic population. Such identifications should be accompanied by a statement of confidence—e.g., node support in a phylogenetic analysis and caveats in relation to the breath of sampling in the reference database (e.g., whale forensics; Palumbi and Cipriano 1998).

DNA-led species discovery is more contentious, but again is not new. In animals, inclusion of mtDNA evidence in biogeographic and systematic analyses often reveals unexpected diversity or discordance with morphology, which then prompts re-evaluation of morphological and ecological characteristics and, if warranted, taxonomic revision. But, despite recent proposals (Wiens and Penkrot 2002; Hebert et al. 2004), it does not follow that mtDNA divergence should be a primary criterion for recognizing species boundaries (see also Sites and Marshall 2003). Potential limitations of using mtDNA to infer species boundaries include retention of ancestral polymorphism, male-biased gene flow, selection on any mtDNA nucleotide (as the whole genome is one linkage group), introgression following hybridization, and paralogy resulting from transfer of mtDNA gene copies to the nucleus. These are acknowledged by Hebert et al. (2004) and well documented in the literature (Bensasson et al. 2001; Ballard and Whitlock 2004), including that on birds (Degnan 1993; Quinn and White 1987; Lovette and Bermingham 2001; Weckstein et al. 2001). More specifically, using some level of mtDNA divergence as a yardstick for species boundaries ignores the low precision with which coalescence of mtDNA predicts phylogenetic divergence at nuclear genes (Hudson and Turelli 2003). An additional problem with focusing on mtDNA (or any other molecular) divergence as a primary criterion for recognizing species is that it will lead us to overlook new or rapidly diverged species, such as might arise through divergent selection or polyploidy, and thus to conclude that speciation requires long-term isolation. For example, a recent mtDNA analysis of North American birds (Johnson and Cicero 2004) showed that numerous avian species have low divergences and that speciation can occur relatively rapidly under certain circumstances. We contend, therefore, that whereas divergent or discordant mtDNA sequences might stimulate taxonomic reassessment based on nuclear genes as well as morphology, ecology, or behavior, mtDNA divergence is neither necessary nor sufficient as a criterion for delineating species. This view accords with existing practice: taxonomic splits in North American birds typically are based on multiple lines of biological evidence, e.g., morphological and vocal differences as well as genetic data (American Ornithologists' Union 1998).

We turn now to the core of Hebert et al.'s paper—COI sequencing of a substantial sample of North American birds (260 of 667 species) and its validity as a test of the barcoding concept. Their aim is to test “the correspondence between species boundaries signaled by COI barcodes and those established by prior taxonomic research.” North American birds are an interesting choice because their species-level taxonomy is relatively well resolved and there has been extensive previous analysis of levels of mtDNA sequence divergence within and among described species (Klicka and Zink 1997; Avise and Walker 1998; Johnson and Cicero 2004). Herbert et al. (2004) found differences in COI sequences “between closely related species” that were 19–24 times greater in magnitude than the differences within species (7.05%–7.93% versus 0.27%–0.43%, respectively). From these data, they conclude that most North American bird species can be discriminated via molecular diagnosis of individuals and propose a “standard sequence threshold” of ten times the mean intraspecific variation (yielding a 2.7% threshold in birds) to flag genetically divergent taxa as “provisional species.” Thus, their analysis seeks to address both potential applications of DNA barcoding.

Although Herbert et al. sampled a large number of species, a true test of the precision of mtDNA barcodes to assign individuals to species would include comparisons with sister species—the most closely related extant relatives. This would require that all members of a genus be examined, rather than a random sample of imprecisely defined close relatives, and that taxa be included from more than one geographic region. Johnson and Cicero (2004) showed the importance of comparing sister species when examining genetic divergence values in North American birds, with results that contrast strongly with those of Hebert et al. as well as previous studies (e.g., Klicka and Zink 1997). For 39 pairs of avian sister species, mtDNA sequence divergences ranged from 0.0% to 8.2%, with an average of 1.9% (cf. 7% to 8% among closely related species in Hebert et al.). Of these, 29 pairs (74%) are at or below the 2.7% threshold proposed by Herbert et al. and thus would not be recognized as species despite biological differences. Moreover, although only a few of these 39 pairs (see Table 1 in Johnson and Cicero [2004]) had sufficient sampling to assess intraspecific variation in mtDNA sequences, these typically showed paraphyly in mtDNA haplotypes. Therefore, there are still too few cases with adequate sampling of intraspecific diversity for sister species pairs to know how common paraphyly is, although a recent meta-analysis found that 17% of bird species deviated from mtDNA monophyly (Funk and Omland 2003). Collectively, these observations cast doubt on the precision of DNA barcoding for allocating individuals to previously described avian species.

Empidonax flycatchers, which are renowned for their morphological similarity and could thereby benefit from DNA-based identification tools, provide an example of the importance of a more detailed analysis. A complete molecular phylogeny for this group (Johnson and Cicero 2002) yielded distances between four pairs of sister species that ranged from 0.7% (E. difficilis versus E. occidentalis) to 4.6% (E. traillii versus E. alnorum); notably, the genetic distance between mainland and island populations of E. difficilis (E. d. difficilis and E. d. insulicola, 0.9%) was greater than that between sister species (Johnson and Cicero 2002). Herbert et al.'s analysis included only two species of Empidonax (E. traillii and E. virescens), which are not sisters but members of divergent clades. Because E. virescens is genetically distant from all other species of Empidonax (10.3% to 12.5% uncorrected distance; Johnson and Cicero 2002), its comparison with E. trailli therefore inflates estimates of interspecific distances within the genus.

Another key point of Hebert et al.'s analysis was to estimate levels of intraspecific diversity. For 130 species of the 260 examined, more than two individuals were sequenced (*n* = 2 to 12 individuals per species, mean = 2.4), and pooled pairwise genetic distances were found to be uncorrelated with geographic distances, leading Hebert et al. to conclude that “high levels of intraspecific divergence in COI in North American birds appear uncommon.” However, this makes the assumption that there is a common underlying pattern of phylogeographic structure, which is unlikely for North American birds (Zink 1996, Zink et al. 2001). If there is significant variation, assessment of intraspecific diversity can be based on a small sample of individuals only if individuals are sampled across existing population subdivisions for which geography and phenotypic variation are reasonable initial surrogates.

The analyses presented by Hebert et al. will certainly stimulate further debate (a reply by Hebert et al. to the present letter is posted at http://www.barcodinglife.com), but, for the reasons outlined here, they are not yet a definitive test of the utility of DNA barcoding for either diagnosis of individuals or discovery of species. We also question whether the results for North American birds can be extrapolated to the tropics, where DNA barcoding could have maximum value. In general, among-population sequence divergence increases with decreasing latitude, even excluding previously glaciated regions (Martin and MacKay 2004), and studies of intraspecific genetic diversity in Neotropical birds have revealed a higher level of phylogeographic subdivision compared to temperate species (Remsen 1997, Lovette and Bermingham 2001). Thus, the general utility of mtDNA barcoding across different biogeographic regions—and between resident versus migratory taxa—requires further scrutiny.

There is little doubt that large-scale and standardized sequencing, when integrated with existing taxonomic practice, can contribute significantly to the challenges of identifying individuals and increasing the rate of discovering biological diversity. But to determine when and where this approach is applicable, we now need to discover the boundary conditions. The real challenge lies with tropical taxa and those with limited dispersal and thus substantial phylogeographic structure. Such analyses need to be taxonomically broad and need to extend beyond the focal geographic region to ensure that potential sister taxa are evaluated and can be discriminated. There is also the need to examine groups with frequent (possibly cryptic) hybridization, recent radiations, and high rates of gene transfer from mtDNA to the nucleus. Only then will the skeptics be satisfied.

## Abbreviations

COI: cytochrome *c* oxidase I
